# A programme to improve quality of care for patients with chronic diseases, Kazakhstan

**DOI:** 10.2471/BLT.18.227447

**Published:** 2019-01-27

**Authors:** Benjamin TB Chan, Chris Rauscher, Arman M Issina, Laura H Kozhageldiyeva, Dametken D Kuzembaeva, Connie L Davis, Helena Kravchenko, Michael Hindmarsh, Jessie McGowan, Gulnara Kulkaeva

**Affiliations:** a Institute for Health Policy, Management and Evaluation, University of Toronto, 155 College St., 4th floor, Toronto, M5T 3M6, Canada.; b Department of Medicine, University of British Colombia, Vancouver, Canada.; c Deceased, formerly Canadian Society for International Health, Ottawa, Canada.; d Canadian Society for International Health, Ottawa, Canada.; e Polyclinic #2, Pavlodar, Kazakhstan.; f Centre for Collaboration, Motivation and Innovation, Hope, Canada.; g North Kazakhstan oblast clinic, Petropavlovsk, Kazakhstan.; h School of Epidemiology and Public Health, University of Ottawa, Ottawa, Canada.; i Kazakhstan Ministry of Health and Social Affairs, Nursultan, Kazakhstan.

## Abstract

**Objective:**

To evaluate the effect of a disease management programme in Kazakhstan on quality indicators for patients with hypertension, diabetes and chronic heart failure.

**Methods:**

A supportive, interdisciplinary, quality improvement programme was implemented between November 2014 and November 2015 at seven polyclinics in Pavlodar and Petropavlovsk. Quality improvement teams were established at each clinic and quality improvement tools were introduced, including patient flowsheets, decision support tools, patient registries, a patient recall process, support for patient self-management and patient follow-up with intensity adjusted for level of disease control. Clinic teams met for four 3-day interactive learning sessions within 1 year, with additional coaching visits. Implementation was managed by five local coordinators and consultants trained by international consultants. National and regional steering committees monitored progress.

**Findings:**

Between July and October 2015, the proportion of hypertensive patients with the recommended blood pressure increased from 24% (101/424) to 56% (228/409). Among patients with diabetes, the proportion who recently underwent eye examinations increased from 26% (101/391) to 71% (308/433); the proportion who had their low-density lipoprotein cholesterol measured increased from 57% (221/391) to 85% (369/433); and the proportion who had their albumin : creatinine ratio measured increased from 11% (44/391) to 49% (212/433). The proportion of chronic heart failure patients who underwent echocardiography rose from 91% (128/140) to 99% (157/158). All patients set themselves self-management goals.

**Conclusion:**

This intensive, supportive, multifaceted programme was associated with significant improvements in quality of care for patients with chronic disease. Further investment in coaching capacity is needed to extend the programme nationally.

## Résumé

**Objectif:**

Évaluer l'impact d'un programme de gestion des maladies au Kazakhstan sur des indicateurs de qualité chez des patients souffrant d'hypertension, de diabète et d'insuffisance cardiaque chronique.

**Méthodes:**

Un programme de soutien interdisciplinaire pour l'amélioration de la qualité a été mis en œuvre entre novembre 2014 et novembre 2015 dans sept polycliniques à Pavlodar et Petropavlovsk. Des équipes spécialisées ont été créées dans chaque établissement et des outils d'amélioration de la qualité ont été instaurés, parmi lesquels des diagrammes de flux de patients, des dispositifs d'aide à la prise de décision, des registres de patients, un processus de rappel des patients, ainsi qu'une assistance pour l'autogestion et le suivi des patients dont l'intensité est ajustée en fonction du degré de contrôle requis. Les équipes cliniques se sont rencontrées à quatre reprises durant l'année pour participer à des sessions d'apprentissage de trois jours chacune, agrémentées de visites d'encadrement complémentaires. La mise en œuvre a été effectuée par cinq coordinateurs et consultants locaux formés par des consultants internationaux. Des comités directeurs nationaux et régionaux se sont chargés de suivre les progrès accomplis.

**Résultats:**

Entre juillet et octobre 2015, la part de patients hypertendus affichant le niveau de tension artérielle recommandé est passée de 24 % (101/424) à 56 % (228/409). Pour les patients souffrant de diabète, la proportion de patients testés pour un taux de cholestérol lié au lipoprotéines de basse densité est passé de 57% (221/391) à 85% (369/433); la proportion de patients testés pour le ratio albumine-créatinine est passé de 11% (44/391) à 49% (212/433); et la part des patients qui ont récemment subi un examen ophtalmologique a augmenté de 26% (101/391) à 71% (308/433).  La proportion de patients souffrant d’insuffisance cardiaque chronique qui se sont soumis à une échocardiographie auparavant a augmenté, passant de 91% (128/140) à 99% (157/158). Tous les patients se sont fixé des objectifs d'autogestion.

**Conclusion:**

Ce programme multiforme de soutien intensif a entraîné une nette amélioration de la qualité des soins aux patients souffrant de maladies chroniques. Des investissements supplémentaires dans les capacités d'encadrement sont nécessaires pour déployer le programme à l'échelle nationale.****

## Resumen

**Objetivo:**

Evaluar el efecto de un programa de gestión de enfermedades en Kazajstán sobre los indicadores de calidad de los pacientes con hipertensión, diabetes e insuficiencia cardíaca crónica.

**Métodos:**

Entre noviembre de 2014 y noviembre de 2015 se llevó a cabo un programa de apoyo, interdisciplinario y de mejora de la calidad en siete policlínicos de Pavlodar y Petropavlovsk. Se establecieron equipos de mejora de la calidad en cada clínica y se incorporaron instrumentos de mejora de la calidad, como hojas de evolución de pacientes, instrumentos de apoyo a la toma de decisiones, registros de pacientes, un proceso de llamadas para recordar citas a los pacientes, apoyo a la autogestión de los pacientes y seguimiento de los pacientes con una intensidad ajustada al nivel de control de la enfermedad. Los equipos clínicos se reunieron en cuatro sesiones de aprendizaje interactivo de tres días en el plazo de un año, con visitas adicionales de entrenamiento. Cinco coordinadores y consultores locales, formados por consultores internacionales, gestionaron la implementación. Los comités directivos nacionales y regionales supervisaron los progresos realizados.

**Resultados:**

Entre julio y octubre de 2015, el porcentaje de pacientes hipertensos con la presión arterial recomendada aumentó del 24 % (101/424) al 56 % (228/409). Entre los pacientes con diabetes, el porcentaje que se sometió recientemente a exámenes oculares aumentó del 26 % (101/391) al 71 % (308/433); el porcentaje a los que se les midió el colesterol de lipoproteína de baja densidad aumentó del 57 % (221/391) al 85 % (369/433); y el porcentaje a los que se les midió la proporción albúmina/creatinina aumentó del 11 % (44/391) al 49 % (212/433). El porcentaje de pacientes con insuficiencia cardíaca crónica que se sometieron a una ecocardiografía aumentó del 91 % (128/140) al 99 % (157/158). Todos los pacientes se fijaron objetivos de autogestión.

**Conclusión:**

Este programa intensivo, de apoyo y multifacético se asoció con mejoras significativas en la calidad de la atención de los pacientes con enfermedades crónicas. Se necesita una inversión adicional en la capacidad de entrenamiento para ampliar el programa a nivel nacional.

## ملخص

**الغرض:**

تقييم تأثير برنامج إدارة الأمراض في كازاخستان على مؤشرات الجودة للمرضى الذين يعانون من ارتفاع ضغط الدم والسكري وقصور القلب المزمن.

الطريقة تم تنفيذ برنامج داعم متعدد التخصصات لتحسين الجودة بين نوفمبر/تشرين ثان 2014، ونوفمبر/تشرين ثان 2015، في سبع عيادات شاملة في بافلودار وبتروبافلوفسك. تم إنشاء فرق لتحسين الجودة في كل عيادة، كما تم إدخال أدوات لتحسين الجودة، بما في ذلك مستندات تدفق المرضى، وأدوات دعم القرار، وسجلات المرضى، وعملية استدعاء المريض، ودعم الإدارة الذاتية للمريض ومتابعة المريض، مع ضبط الكثافة على مستوى السيطرة على الأمراض. اجتمعت فرق العيادات في أربع جلسات تعلم تفاعلية لمدة 3 أيام خلال عام واحد، مع زيارات تدريب إضافية. تمت أدارة التنفيذ بواسطة خمس منسقين واستشاريين محليين تم تدريبهم بواسطة استشاريين دوليين. وقامت اللجان التنظيمية الوطنية والإقليمية بمراقبة التقدم.

النتائج بين شهري يوليو/تموز وأكتوبر/تشرين أول لعام 2015، فإن نسبة المرضى الذين يعانون من ارتفاع ضغط الدم، ولديهم ضغط الدم الموصى به، قد ارتفعت من 24% (101/424) إلى 56% (228/409). وبين مرضى السكري، فإن النسبة التي خضعت مؤخراً لفحوصات العين قد زادت من 26% (101/391) إلى 71% (308/433)؛ كذلك فإن نسبة الذين يقومون بقياس كوليستيرول البروتين الدهني منخفض الكثافة لديهم، قد زادت من 57% (221/391) إلى 85% (369/433)؛ ونسبة الذين يقومون بقياس نسبة الزلال إلى الكرياتينين لديهم، قد زادت من 11% (44/391) إلى 49% (212/433). ارتفعت نسبة مرضى قصور القلب المزمن الذين خضعوا لتخطيط صدى القلب من 91% (128/140) إلى 99% (157/158). وضع جميع المرضى أنفسهم كأهداف للإدارة الذاتية.

الاستنتاج ارتبط هذا البرنامج المكثف الداعم متعدد الأوجه بتحسينات ملموسة في جودة الرعاية للمرضى الذين يعانون من مرض مزمن. هناك حاجة إلى مزيد من الاستثمار في قدرة التدريب لتوسيع البرنامج على المستوى الوطني.

## 摘要

**目的:**

旨在评估哈萨克斯坦疾病管理项目对高血压、糖尿病和慢性心力衰竭患者护理质量指标的影响。

**方法:**

2014 年 11 月至 2015 年 11 月期间，在巴甫洛达尔和彼得罗巴甫洛夫斯克的七家综合诊所实施了支持性、跨学科的质量改进项目。在每个诊所中都成立了质量改进团队并引入了质量改进工具，包括患者流程图、决策支持工具、患者登记表、患者复诊提醒流程、对患者自我管理以及患者随诊的支持，并根据疾病控制程度来调整强度。诊所团队在一年内举行了四次为期 3 天的互动式学习会议，并进行了额外的指导考察。实施工作由五名当地协调员和经过国际顾问培训的顾问管理。国家和地区指导委员会监测进展。

**结果:**

2015 年 7 月至 10 月期间，血压达到推荐标准的高血压患者的比例从 24% (101/424) 增至 56% (228/409)。在糖尿病患者中，近期接受眼科检查的患者比例从 26% (101/391) 增至 71% (308/433)；接受低密度脂蛋白胆固醇测定的患者比例从 57% (221/391) 增至 85% (369/433)；接受白蛋白/肌酐比值测定的患者比例从 11% (44/391) 增至 49% (212/433)。接受超声心动图检查的慢性心力衰竭患者比例从 91% (128/140) 增至 99% (157/158)。所有患者都为自己设定了自我管理目标。

**结论:**

这个强化支持性多维项目促使慢性病患者的护理质量显著改进。需要进一步加大投资，提高指导能力，在全国范围内推广该项目。****

## Резюме

**Цель:**

Оценить влияние программы управления заболеваний, действующей в Казахстане, на показатели качества медицинской помощи, предлагаемой пациентам с гипертензией, диабетом и хронической сердечной недостаточностью.

**Методы:**

Комплексная междисциплинарная программа, направленная на улучшение качества медицинской помощи, проводилась в семи поликлиниках Павлодара и Петропавловска в период с ноября 2014 года по ноябрь 2015 года. В каждой поликлинике были созданы группы по повышению качества медицинского обслуживания, использовавшие соответствующие технические средства, включая графики приема пациентов, средства поддержки принятия решений, реестры пациентов, процесс повторного вызова пациентов, обучение пациентов методам самопомощи и последующее наблюдение пациентов с частотой, зависящей от уровня контроля заболевания. В течение года группы специалистов поликлиники четырежды собирались для проведения 3-дневных курсов интерактивного обучения и получали дополнительное индивидуальное обучение. Внедрением программы занимались пять местных координаторов и консультантов, прошедших международное обучение. За ходом выполнения программы следили национальный и региональный руководящие комитеты.

**Результаты:**

В период между июлем и октябрем 2015 года доля пациентов, страдающих гипертензией, у которых отмечалось рекомендованное кровяное давление, выросла с 24% (101 из 424) до 56% (228 из 409). Среди пациентов с диабетом доля лиц, недавно прошедших обследование у окулиста, возросла с 26% (101 из 391) до 71% (308 из 433); доля тех, кому определяли уровень холестерина липопротеинов низкой плотности, увеличилась с 57% (221 из 391) до 85% (369 из 433); а доля тех, кому измеряли соотношение альбумина к креатинину, возросла с 11% (44 из 391) до 49% (212 из 433). Среди пациентов с хронической сердечной недостаточностью доля пациентов, прошедших эхокардиографию, увеличилась с 91% (128 из 140) до 99% (157 из 158). Все пациенты поставили себе цели по изучению методов самопомощи.

**Вывод:**

Интенсивная, комплексная, разноплановая программа была связана со значительным улучшением качества медицинской помощи пациентам с хроническими заболеваниями. Для распространения программы в национальном масштабе нужны дальнейшие инвестиции в обучающую деятельность.

## Introduction

Kazakhstan has a high rate of premature death from noncommunicable diseases; in 2012, it was 648 deaths per 100 000 adults compared with an average of 395 per 100 000 in the World Health Organization’s (WHO’s) European Region.^1^^,^^2^ Many deaths could be prevented by applying evidence-based practices for treatment, monitoring and promoting healthy behaviour. Previously, no system for routinely monitoring adherence to best practice existed in the country and surveys have identified major gaps in treatment. For example, in 2010, only 27% of 1799 hypertensive patients surveyed were taking prescribed medications daily.^3^ Moreover, in one city, only 34% (119/350) of hypertensive patients had their blood pressure controlled^4^ and only 28% (33/119) of patients with diabetes had adequate fasting plasma glucose levels.^5^

Combating noncommunicable diseases depends on improving the quality of care. A 2018 report by the *Lancet* Global Health Commission estimated that 8 million lives are lost globally each year because of poor care quality. As in Kazakhstan, health-care providers in many low- and middle-income countries follow guidelines on common medical conditions less than half the time.^6^ Another 2018 report notes the proportion of hypertensive patients treated adequately varied from 7 to 61% globally.^7^

Better quality depends on a strong primary care system, where most treatment, monitoring and counselling takes place. Historically, primary care has been weak in countries of the former Soviet Union, where care was strongly specialist-based.^8^ In Kazakhstan, change began in 2004 when the State Health Care Reform and Development Program prioritized primary care and decentralized health services.^8^ Between 2008 and 2015, the country embarked on the ambitious Health Sector Technology Transfer and Institutional Reform Project, financed by the World Bank.^9^ The project expanded universal health insurance, accreditation programmes, information systems and clinical practice guidelines.

The aim of this paper was to describe the results of a disease management programme established in the last year of the 8-year project. The programme set out to improve process and outcome measures for diabetes, hypertension and chronic heart failure in primary care by using quality improvement techniques to maximize the adoption of clinical practice guidelines. Previously, such techniques have been used successfully in high-income countries for chronic disease management in primary care. For example, the Health Disparities Collaboratives in the United States of America improved the quality of diabetic care among vulnerable populations.^10^ This paper provides new information on how quality improvement techniques can be applied in a middle-income country with a distinct culture, governance system and primary care infrastructure.

## Methods

In Kazakhstan, primary care is provided through polyclinics by specialists, therapists (i.e. internists), general practitioners, nurses, psychologists, social workers and health educators. Laboratory and diagnostic imaging services are also available on site. Polyclinics are publicly funded and provide essential services for free within their catchment areas. Urban polyclinics report to the health department of the *oblast* (i.e. subnational region), which in turn reports to the national health ministry.

We investigated the effect of the initial design and testing phase of the disease management programme, from November 2014 to November 2015. The programme was implemented in seven large urban polyclinics in Pavlodar and Petropavlovsk (population 308 000 and 195 000, respectively). Three clinic teams worked on diabetes, three worked on hypertension and two worked on chronic heart failure. In one clinic, two disease types were tackled simultaneously. This phase did not include private clinics or public clinics in rural areas, which offer a limited range of services.

### Programme design

To assess quality, countries of the former Soviet Union traditionally relied on clinical protocols, which specified standards for medical practice against which physicians were audited and sanctioned if found noncompliant.^11^ This approach assumed that poor care quality was due to a lack of effort that could be remedied by punishment and ignored the fact that poor quality was often due to systemic obstacles. In contrast, the disease management programme adopted a supportive, team-based, multifaceted approach to quality improvement that aimed to help clinic teams address the root causes of poor care in an environment that emphasized learning, analysis and improving work processes. The programme used the Chronic Care Model as a blueprint for designing a primary health-care system to manage chronic diseases and included the following components: (i) decision support tools for clinicians; (ii) an information system; (iii) care delivery system design; and (iv) patient self-management.^12^

Decision support tools are intended to remind clinicians of the actions to be taken in different situations. They address the problem that guidelines are often complex and easy to forget and that some health-care providers may not be aware of their contents.^13^ The main tool was a flowsheet – a one-page document included in each patient’s chart to remind staff which tasks should be performed and documented at each clinic visit. The document also recorded clinical data, such as blood pressure, laboratory measurements and health-related behaviours. A flowsheet was developed for each targeted condition based on international examples. Other tools included simple, one-page algorithms for diagnosis or selecting treatment and checklists for the tests required. These tools were user-friendly alternatives to clinical protocols, which can be lengthy, legalistic and dense. All tools were approved by a clinical advisory group.

The clinical information system comprised a patient registry, which addressed the problem that health-care providers may be unaware of gaps in care that need attention. At each patient encounter, clinic staff entered data required by the flowsheet into an Excel database (Microsoft Corporation, Redmond, USA), which automatically calculated values for quality indicators. Staff could then review areas of weakness monthly and target them for improvement. The registry also reported changes in indicators over time, which helped in monitoring the programme’s impact.

A care delivery system was designed to ensure key processes were performed consistently. The system addressed the problem that the steps involved in delivering care are often poorly coordinated or implemented, or inefficient. There were three process improvements: (i) a recall process was created to ensure patients overdue for follow-up or a test returned to the clinic; (ii) patient segmentation was introduced to group patients by level of disease control; and (iii) structured visits were introduced. Box 1 describes these approaches in more detail.

Box 1 **Improvements to optimize quality of care of chronic diseases, Kazakhstan, 2015**

**A recall process**

This process helped to ensure that patients overdue for follow-up or a test returned to the clinic. Practice guidelines recommend patients with diabetes undergo measurement of HbA1c every 6 months and LDL cholesterol measurement every 12 months. The patient registry was designed to generate recall lists of patients overdue for follow-up or a test. Each polyclinic was required to refine its recall process. Typically, polyclinics assigned one individual to review recall lists weekly and ensure patients were phoned or otherwise encouraged to return to the clinic.

**Patient segmentation**

This process aimed to group patients by level of disease control. For example, diabetes patients with a blood pressure and HbA1c and LDL cholesterol levels within desired limits were deemed optimal. Those with an HbA1c level above 7% were suboptimal and an HbA1c level over 9% indicated poor control. Each clinic developed standard processes for determining how frequently and intensely each patient group should be followed up. For example, a patient with well controlled hypertension could be seen every 6 months, whereas one with a systolic and diastolic blood pressure above 160 and 100 mmHg, respectively, could be seen monthly until control was achieved. Previously in Kazakhstan, all patients were seen monthly. The aim of segmentation was to improve efficiency by reducing unnecessary visits for healthier patients and reallocate staff time to those who needed more attention.

**Structured visits**

Clinic teams were encouraged to identify all tasks included in follow-up assessments, to assign tasks to different team members, to consider shifting tasks between team members (e.g. from a specialist to a primary care physician) to improve efficiency and to develop a routine to avoid omitting tasks by mistake.

HbA1c: glycosylated haemoglobin; LDL: low-density lipoprotein.

The program introduced support for patient self-management, an approach which helps patients manage their condition themselves. Research shows that patients engaged in their own care who understand their condition and know how to modify unhealthy behaviour benefit most from improved clinical care.^14^ Clinic staff learned how to shift from simply providing information to patients or using scare tactics to induce change to, instead, engaging in supportive dialogue. Staff also learned to coach patients to set small, but realistic and specific goals and to help them make several small changes that could gradually strengthen their self-confidence.

The programme was consistent with the three pillars of WHO’s framework on quality in primary health care: (i) empowered people and engaged communities; (ii) multisectoral policy and action for health; and (iii) health services that prioritize the delivery of high-quality primary care.^15^ The first pillar was addressed by the programme’s patient self-management component. The second was addressed by a concurrent project funded by the World Bank, which aimed to expand health insurance coverage, introduce accreditation and provide financial incentives for good performance. The third was addressed by the programme’s decision support tools, performance feedback and process improvements.

### Implementation

**Fig. 1:**
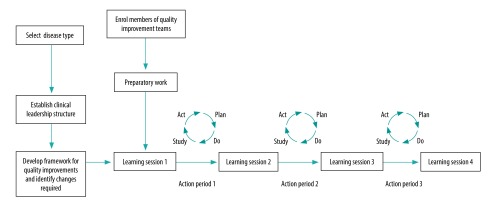
Breakthrough Series Collaborative model used in the Kazakhstan disease management programme, 2015

Notes: The diagram was adapted from the Institute for Healthcare Improvement.^16^ Interdisciplinary quality improvement teams from each pilot site attended quarterly learning sessions and were supported in between sessions by email, visits from consultants and phone conferences. Teams also started reported data each month midway into the collaborative.

We emphasized group learning over multiple encounters instead of traditional lecture-style teaching by using the Breakthrough Series Collaborative model developed for multisite quality improvement initiatives.^16^ Clinic teams attended four 3-day learning sessions in Pavlodar or Petropavlovsk to receive training from international consultants on implementing quality improvements. Each city had a regional coordinator (a physician with management experience) who worked with the polyclinics and was also trained by the international consultants. Skills, such as support for patient self-management, were taught by studying clinical cases and role-playing. Before each session, teams were assigned preparatory work and sessions were used to report progress, identify obstacles and brainstorm solutions with other participating teams. Between learning sessions, the international consultants made coaching visits and participants conducted Plan–Do–Study–Act cycles to test and customize quality improvement tools from elsewhere and adapt them for local use (Fig. 1).

A formal leadership structure was established at different levels. Each polyclinic identified a clinical coordinator (i.e. team leader) and formed an interdisciplinary quality improvement team. The health ministry appointed a national coordinator and the two regional coordinators noted above. Progress across all sites was reviewed by a national steering committee and, at the regional level, by regional steering committees.

The core implementation team comprised five international consultants (two full-time equivalents) and two full-time local consultants and was active over 13 months. The programme’s costs included staff remuneration, the cost of office space, room rental, printed material and translations, and travel costs for meetings within the country and for six missions by international consultants. There were substantial in-kind contributions of personnel time from health ministry staff and other key stakeholders, which included time for participating in steering committees and clinical advisory groups. One full-time staff member from the health ministry was designated the programme liaison officer.

### Evaluation

Our investigation employed a quasi-experimental study design, where differences in quality indicators from before to after the intervention were examined for a single study group. Clinic teams submitted data monthly from July to October 2015. During this time, teams implemented programme components, such as recall processes, patient segmentation and support for patient self-management. Differences between the two periods were assessed using a two-tailed *t*-test for the difference between proportions. Quality indicators were selected for diabetes, hypertension and chronic heart failure by reviewing indicators used in other countries or recommended by clinical guidelines (Table 1). All indicators were approved by a national clinical advisory group. Indicators included both process measures (e.g. adoption of guideline recommendations on the use of drugs and tests, and on follow-up) and outcome measures (e.g. blood pressure, blood sugar and cholesterol levels).

**Table 1:** Effect of a disease management programme on the quality of chronic disease care, Kazakhstan, 2015

Disease and quality criterion^a^	No. of patients assessed					No. of patients who met criterion (%)				*P*^c^
	July^b^	August	September	October		July^b^	August	September	October	
**Hypertension**										
Blood pressure checked at last polyclinic visit^d^	315	423	415	409		256 (81)	365 (86)	388 (93)	391 (96)	< 0.001
Systolic/diastolic blood pressure < 140/90 mmHg^e^	424	423	415	409		101 (24)	178 (42)	197 (47)	228 (56)	< 0.001
**Diabetes**										
Eye examination in past year^d^	391	317	445	433		101 (26)	76 (24)	181 (41)	308 (71)	< 0.001
LDL cholesterol measured in past year^d^	391	317	445	433		221 (57)	211 (67)	342 (77)	369 (85)	< 0.001
Albumin : creatinine ratio measured in past year^d^	391	317	445	433		44 (11)	107 (34)	131 (29)	212 (49)	< 0.001
HbA1c measured in past 6 months^d^	391	317	445	433		282 (72)	188 (59)	327 (73)	326 (75)	0.23
Foot examination in past year^d^	391	317	445	433		261 (67)	192 (61)	320 (72)	305 (70)	0.21
HbA1c level < 7%^e^	282	188	327	326		163 (58)	115 (61)	182 (56)	182 (56)	0.37
Systolic/diastolic blood pressure < 140/90 mmHg^e^	391	317	445	433		225 (58)	179 (56)	246 (55)	246 (57)	0.39
LDL cholesterol level < 2.5 mmol/L^e^	221	211	342	369		59 (27)	50 (24)	74 (22)	64 (17)	0.01
**Chronic heart failure**										
Underwent echocardiography^d^	140	162	162	158		128 (91)	144 (89)	161 (99)	157 (99)	< 0.001

HbA1c: glycosylated haemoglobin; LDL: low-density lipoprotein.

^a^ Quality of care indicators were the percentage of patients who satisfied each criterion.

^b^ The first time at which validated data were available from participating sites. The quality improvement programme was initiated between January and June 2015.

^c^ We used two-tailed *t*-tests for differences in proportions to calculate if there was a statistical difference between patients meeting the criterion in July compared with October.

^d^ Process indicator.

^e^ Outcome indicator.

## Findings

Learning sessions began in January 2015, indicators and flowsheets were established by March 2015 and the patient registry became operational by June 2015. All learning sessions between January and October 2015 included training on patient self-management.

**Fig. 2:**
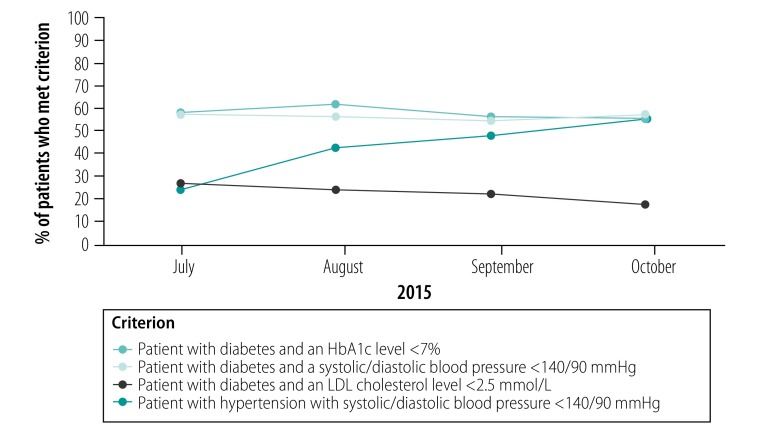
Change in care quality outcome indicators, disease management programme, Kazakhstan, 2015

**Fig. 3:**
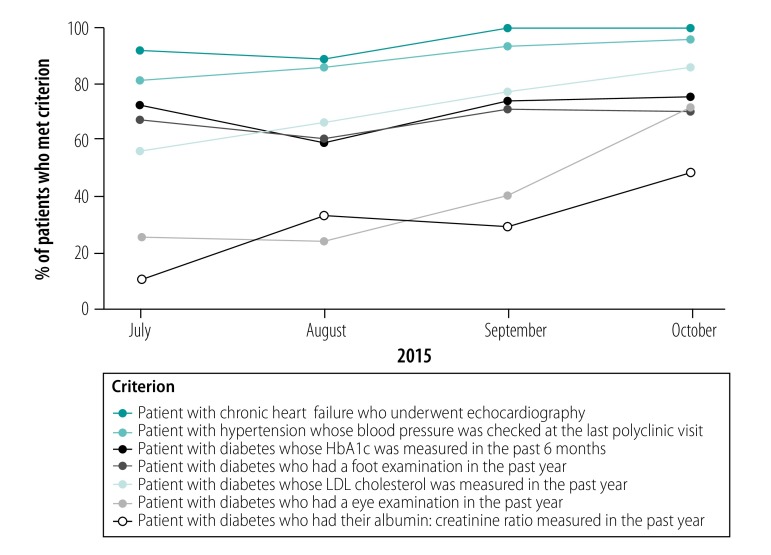
Change in care quality process indicators, disease management programme, Kazakhstan, 2015

Between July and October 2015, the proportion of hypertensive patients whose blood pressure was under control increased significantly (Table 1 and Fig. 2), as did the proportion whose blood pressure was checked at the last clinic visit (Fig. 3). There were also significant increases in the proportion of patients with diabetes who underwent low-density lipoprotein (LDL) cholesterol and albumin: creatinine ratio assessment and had eye examinations in the past year. However, there was no significant change in foot examinations or regular glycosylated haemoglobin (HbA1c) measurement. The proportion of patients with good control of LDL cholesterol (i.e. under 2.5 mmol/L) decreased significantly from 27% (59/221) to 17% (64/369) but there was no significant change in the proportion with good glucose control (i.e. an HbA1c level under 7%) or with a systolic and diastolic blood pressure under 140 mmHg and 90 mmHg, respectively. The proportion of patients with chronic heart failure who underwent echocardiography increased significantly from 91% (128/140) to 99% (157/158). All patients had self-management goals documented and 223 health-care providers underwent basic training on patient self-management. All seven polyclinics achieved a significant improvement in at least one quality indicator.

HbA1c: glycosylated haemoglobin; LDL: low-density lipoprotein.

Note: Validated data were first available from participating sites in July 2015.

HbA1c: glycosylated haemoglobin; LDL: low-density lipoprotein.

Note: Validated data were first available from participating sites in July 2015.

## Discussion

Our investigation showed that the quality improvement tools for chronic disease management developed in high-income settings could be deployed effectively in Kazakhstan. Improvements were achievable despite fewer national resources and the country’s history of limited primary care development. In 2009, only 17% (26 vs 156 per 100 000 population) of physicians in the country were general practitioners and their training programmes were relatively new and focused on knowledge rather than practical skills.^17^

Implementation of the disease management programme was associated with substantial improvements in care quality process measures, such as ensuring patients had recently undergone recommended tests. The recall lists generated by the patient registry were critical for success because they identified patients who needed to return for missed tests. Our observations are consistent with those of the United States’ Health Disparities Collaborative, which found that improvements were greatest for similar quality indicators.^18^

Although quality outcome measures improved for hypertensive patients, similar outcomes did not improve for patients with diabetes over the short-term. However, clinical algorithms were introduced relatively late in the programme and they might have had little impact during the observation period. Moreover, it may require more time to optimize decision-making for more complex treatment decisions. In the Health Disparities Collaborative, early results also showed no improvement in diabetes outcomes,^18^ but some sites demonstrated reductions in HbA1c levels after 4 years of follow-up.^10^

The unusual finding that the proportion of patients with diabetes and an LDL cholesterol level < 2.5 mmol/L decreased probably occurred because increased testing led to greater inclusion of people who were not compliant with the regular visit schedule and who were also probably less likely to comply with dietary recommendations. Members of the national steering committee noted that statins were not free under universal health insurance in Kazakhstan – drug policy may, therefore, need to change. Similarly, the proportion of patients with diabetes whose HbA1c level was measured did not change. Although HbA1c testing is free, some participants noted that budgetary constraints at clinics hindered access to the test. Better planning could improve access.

As the disease management programme had numerous complex components, frequent interactions between international consultants, local coordinators and participants were key to success. These interactions helped clinic teams adapt the quality improvement tools developed elsewhere for local use and assisted in problem-solving. During learning sessions and coaching visits, implementation problems were observed, such as the incomplete use of flowsheets, data entry errors, incorrect techniques in patient self-management discussions and confusion about interpreting guidelines, algorithms or indicators. The traditional learning model of attending a single lecture would probably not have resulted in similar improvements.

The programme’s formal leadership structure provided an accountability mechanism that probably contributed to its success. Progress was reviewed regularly at national and regional steering committees, where problems were identified and solutions discussed. The implementation rate of different quality improvement tools and in the improvements achieved varied between clinics. Later in the programme, clinic teams were asked to present progress reports to their peers during learning sessions and to compare results with each other. This friendly competition helped motivate teams to improve.

Recent WHO recommendations for governments on improving health care emphasize the need for clear strategies on care quality to ensure success and sustainability.^19^ Specifically, WHO guidelines recommend: (i) setting priorities and targets; (ii) engaging stakeholders; (iii) specifying accountability; (iv) identifying indicators; and (v) creating information systems for performance feedback and reporting.^20^ As part of this project which was financed by a World Bank loan, the consulting team in Kazakhstan made recommendations on a national chronic disease strategy that were consistent with WHO’s framework. Stakeholders were engaged in programme design through national and regional steering committees and clinical advisory groups. These committees served as an accountability structure. In addition, it was recommended that accountability be strengthened by extending accreditation criteria to include programme components, such as the use of a patient registry and flowsheets. The quality indicators identified and listed in Table 1 were approved nationally. Regarding information systems, it was recommended that the patient registry be incorporated into future electronic medical records. Finally, financial incentives were introduced to improve primary care performance and recommendations were made on how incentives could be better aligned with the programme’s objectives.

Our quasi-experimental study design was limited by the lack of a control group. However, it is unlikely the large improvements we observed over a short time were due to any factor other than the disease management programme. Moreover, there was no major change in infrastructure, staffing, catchment population or remuneration at pilot sites during the study period. Another limitation was that, although all patients set themselves self-management goals, the quality of the self-management support provided for patients was not assessed. Future studies should include a patient survey to evaluate this support. 

The generalizability of the study’s findings may be limited for two reasons. First, only urban settings were included; implementation of the programme in rural settings with fewer resources may require more support. Second, although Kazakhstan has relatively few primary care physicians, the polyclinic model has strengths that may have contributed to success, such as different health disciplines working together in the same facility. In addition, data literacy was good and most clinics already had data entry staff. Implementation may be harder in settings without equivalent staffing.

Following the success of this pilot, attempts were made to extend the programme throughout Kazakhstan by establishing trainers in each *oblast* to support local polyclinics within the existing health-care system hierarchy. Designing a system to support clinic teams throughout the country proved challenging because the quality improvement model we employed requires high-intensity support and because the number of local coordinators and consultants trained was insufficient for rapid expansion. Currently, a new project financed by a World Bank loan is underway that will increase the number of local facilitators. Our experience confirms that investment in capacity building at the ground level is essential for ensuring sustainability.

The disease management programme in Kazakhstan included a holistic package of interventions, such as patient flowsheets, decision support tools for clinicians, process improvements, support for patient self-management, measurement of quality indicators and performance feedback through an electronic registry. Our pilot study found that significant improvements in care quality could be achieved without an increase in clinic staff. However, success depended critically on intensive coaching and regular support for local clinic teams. The priority for policy-makers who wish to apply this approach in their own countries is to invest in building capacity to provide external support for local clinic teams. Also important are strong leadership, an accountability structure, incentives and continued engagement with stakeholders within the framework of a national plan for improving health-care quality.
